# Correction: Genetic variants influence on the placenta regulatory landscape

**DOI:** 10.1371/journal.pgen.1008118

**Published:** 2019-04-12

**Authors:** Fabien Delahaye, Catherine Do, Yu Kong, Remi Ashkar, Martha Salas, Ben Tycko, Ronald Wapner, Francine Hughes

There are errors in the Funding statement. The correct Funding statement is as follows: This research was supported by the Intramural Research Program of the Eunice Kennedy Shriver National Institute of Child Health and Human Development, National Institutes of Health (Contract Numbers: HHSN275200800013C; HHSN27500006; HHSN275200800003I; HHSN275200800014C; HHSN275200800012C; HHSN275200800028C; HHSN275201000009C). The funders had no role in study design, data collection and analysis, decision to publish, or preparation of the manuscript.
