# Supplementary figures and images for: Genetic variants influence on the placenta regulatory landscape
Source: PLoS Genet. 2018 Nov 19;14(11):e1007785. doi: 10.1371/journal.pgen.1007785 (PMC6277118; doi:10.1371/journal.pgen.1007785)

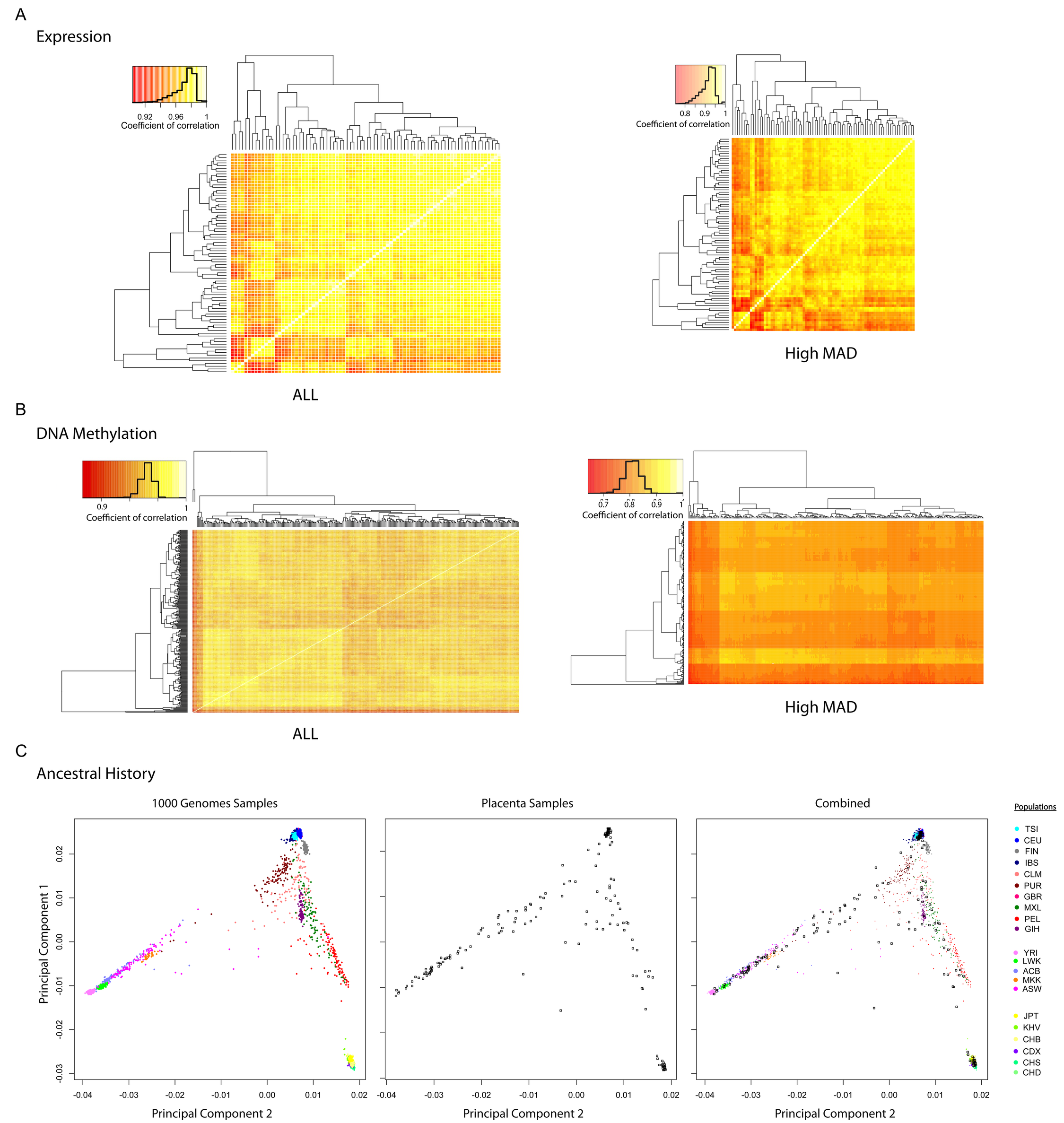

Supplement: S1 Fig — Heatmap representing gene expression correlation across samples for the full dataset (ALL) and highly variable genes defined using MAD (High MAD) (A). Heatmap representing DNA methylation correlation across samples for the full dataset (ALL) and highly variable genes defined using MAD (High MAD) (B). Genetic background analysis using principal component. Each plot represents principal component 1 versus principal component 2 when considering the 1000 genomes samples reference dataset (1000 Genomes Project samples), our placenta cohort (Placenta Samples) and the overlap between the two. Populations are color coded in the reference dataset and black squares represent samples from the placenta cohort (C). (TIF) [file pgen.1007785.s001.tif]

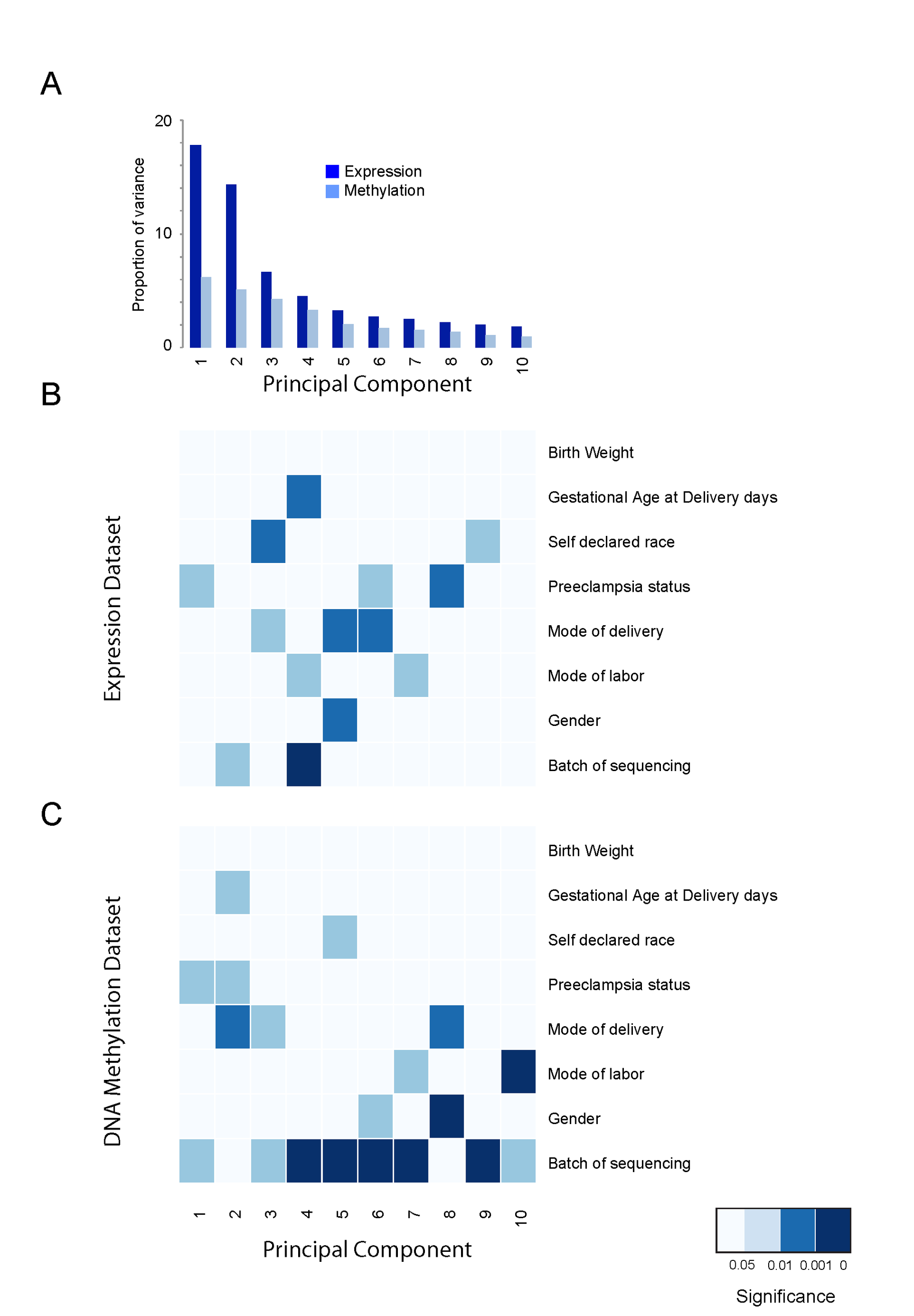

Supplement: S2 Fig — The principal component analysis was run to identify cofounders within the gene expression and DNA methylation datasets. Histogram representing the proportion of variance explains for each principal component from 1 to 10 (A). Association between principal component and the known factor was assessed using linear regression. Heatmap representing the level of significance for the association between a principal component and each factor for gene expression dataset (B) and DNA methylation dataset (C). (TIF) [file pgen.1007785.s002.tif]

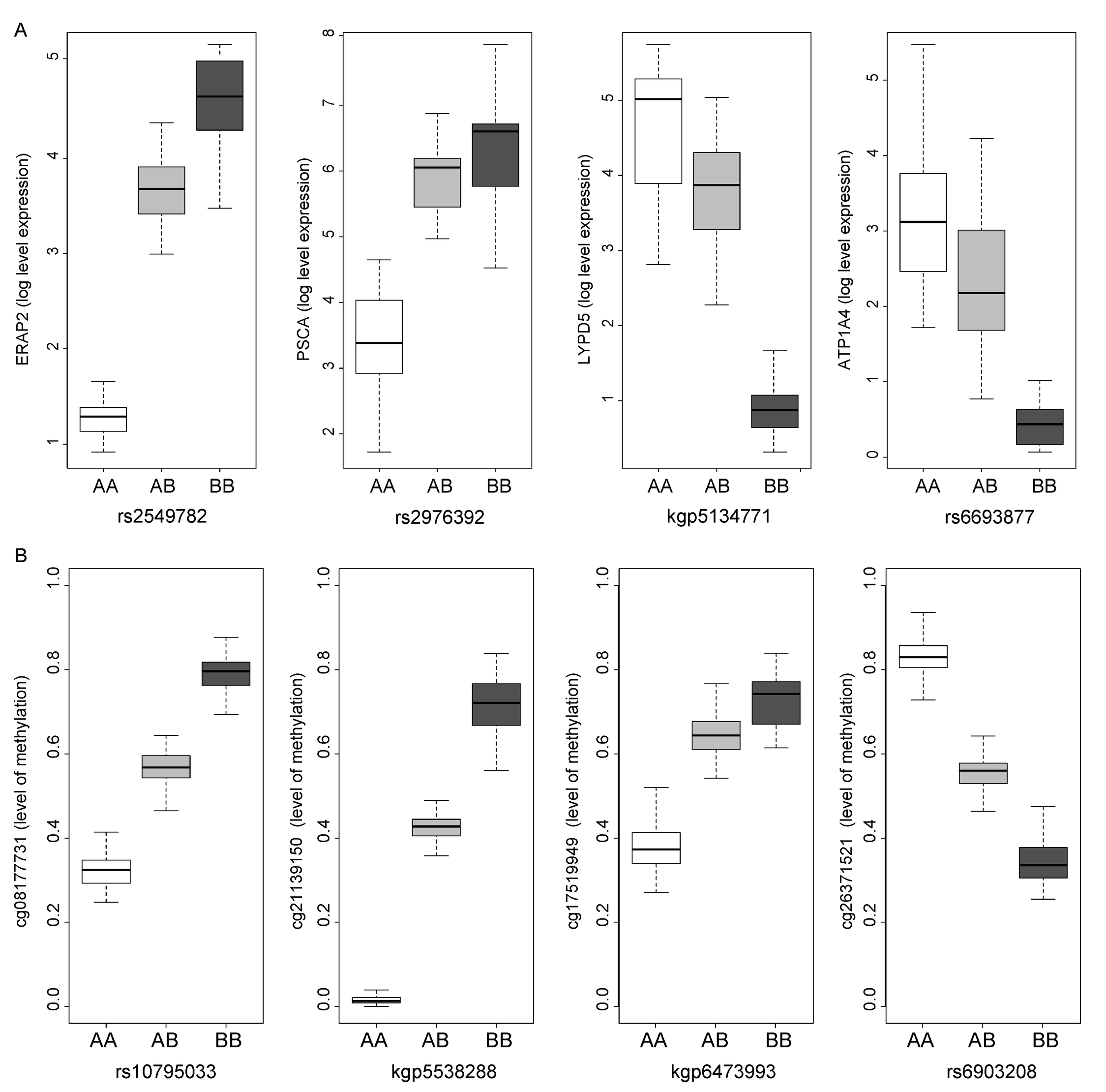

Supplement: S3 Fig — Boxplot representing the profile of expression for each genotype for selected top candidate eQTLs (A). Boxplot representing the profile of methylation for each genotype for selected top candidate mQTLs (B). (TIF) [file pgen.1007785.s003.tif]

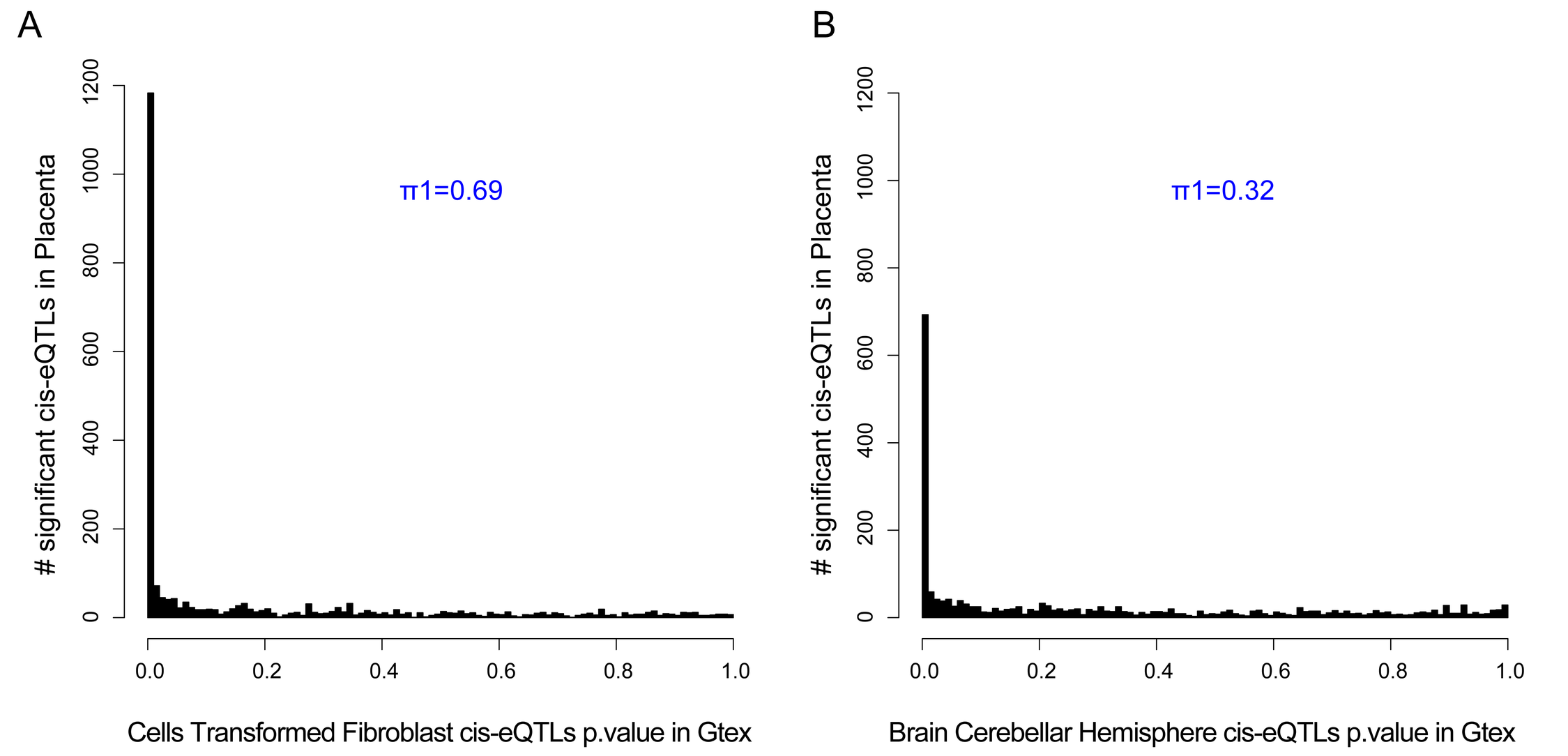

Supplement: S4 Fig — Histogram representing the enrichment for cis-eQTLs from the placenta in transformed fibroblasts from GTEx (A) and in brain cerebellar hemisphere (B). The π1 value represents similarity between tissue ranging from 0 (least similar) to 1 (most similar). (TIF) [file pgen.1007785.s004.tif]

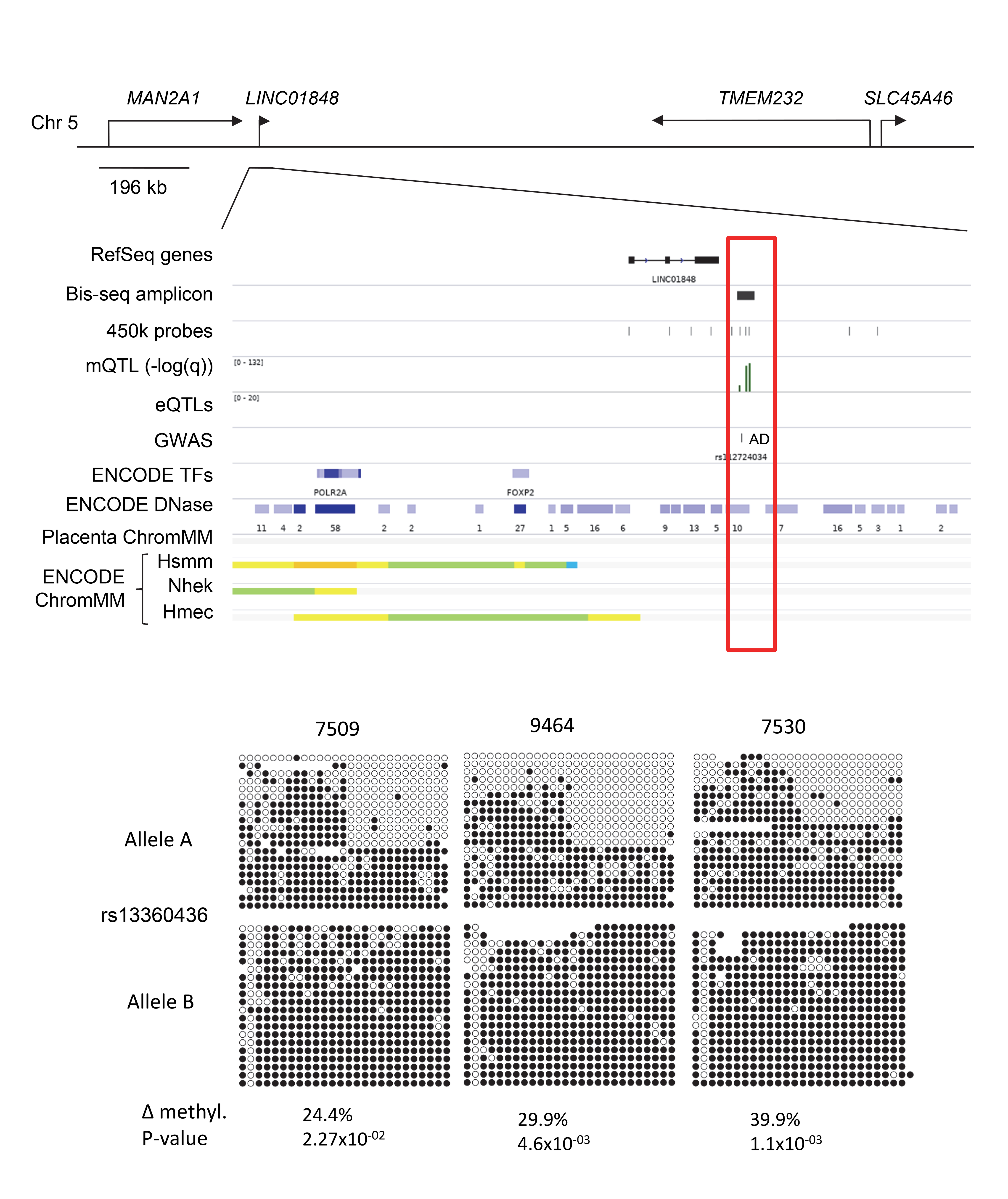

Supplement: S5 Fig — Targeted bis-seq data showing Hap-ASM in LOC01848 region. The bis-seq amplicon covers the mQTL index CpG (cg15548566), as well as contiguous CpGs. This region overlaps with the common SNP, rs13360436 which dictates methylation level with the alternate allele (allele B) being significantly hypermethylated compared to the reference allele (allele A), suggesting the presence of hap-ASM in 11 out of 19 heterozygous samples. The low methylated allele is significantly biased toward allele A (p = 4x10^-06^, using binomial test) which ruled out imprinting. In this region, rs112724034 has been associated with Alzheimer’s disease (AD) [1]. ΔMeth (difference in the percentage of methylation between alleles in heterozygous samples) and Wilcoxon p-values are from bootstrapping. (TIF) [file pgen.1007785.s005.tif]

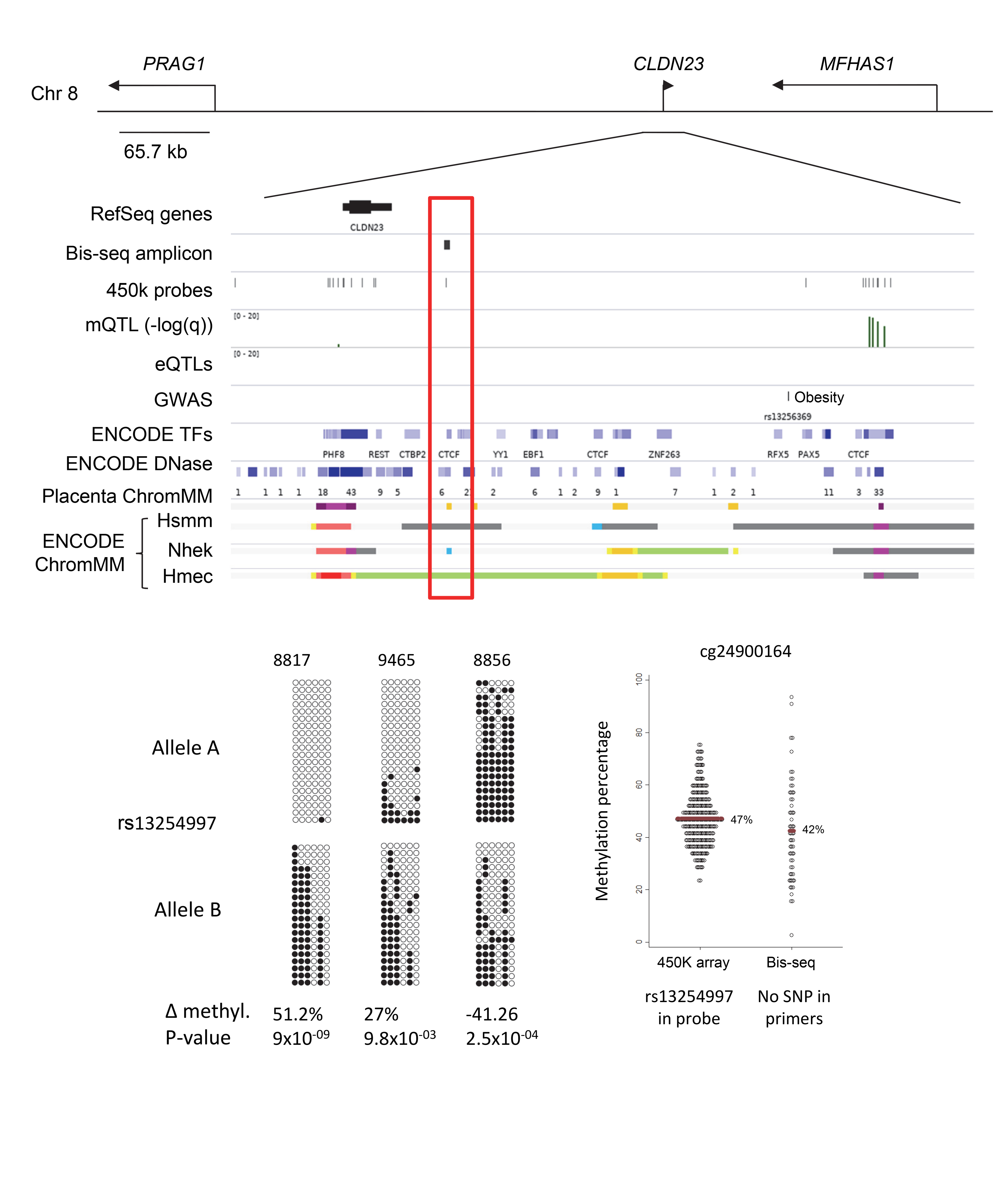

Supplement: S6 Fig — Bis-seq showing hap-ASM in CLDN23 region. cg24900164 was excluded from our main analysis since the probe maps a common non-CT SNP located 7 bp from the index CpG and was therefore not included in our stringent mQTL list. However, targeted bis-seq identified hap-ASM dictated by rs13254997 in 9 out of 20 heterozygous samples (7 positives and 2 negative hap-ASM). In addition, the net methylation estimates from Illumina 450K BeadChips arrays and bis-seq were similar suggesting that the SNP did not affect the probe hybridization. These findings suggest the presence of genuine mQTL at this locus. (TIF) [file pgen.1007785.s006.tif]

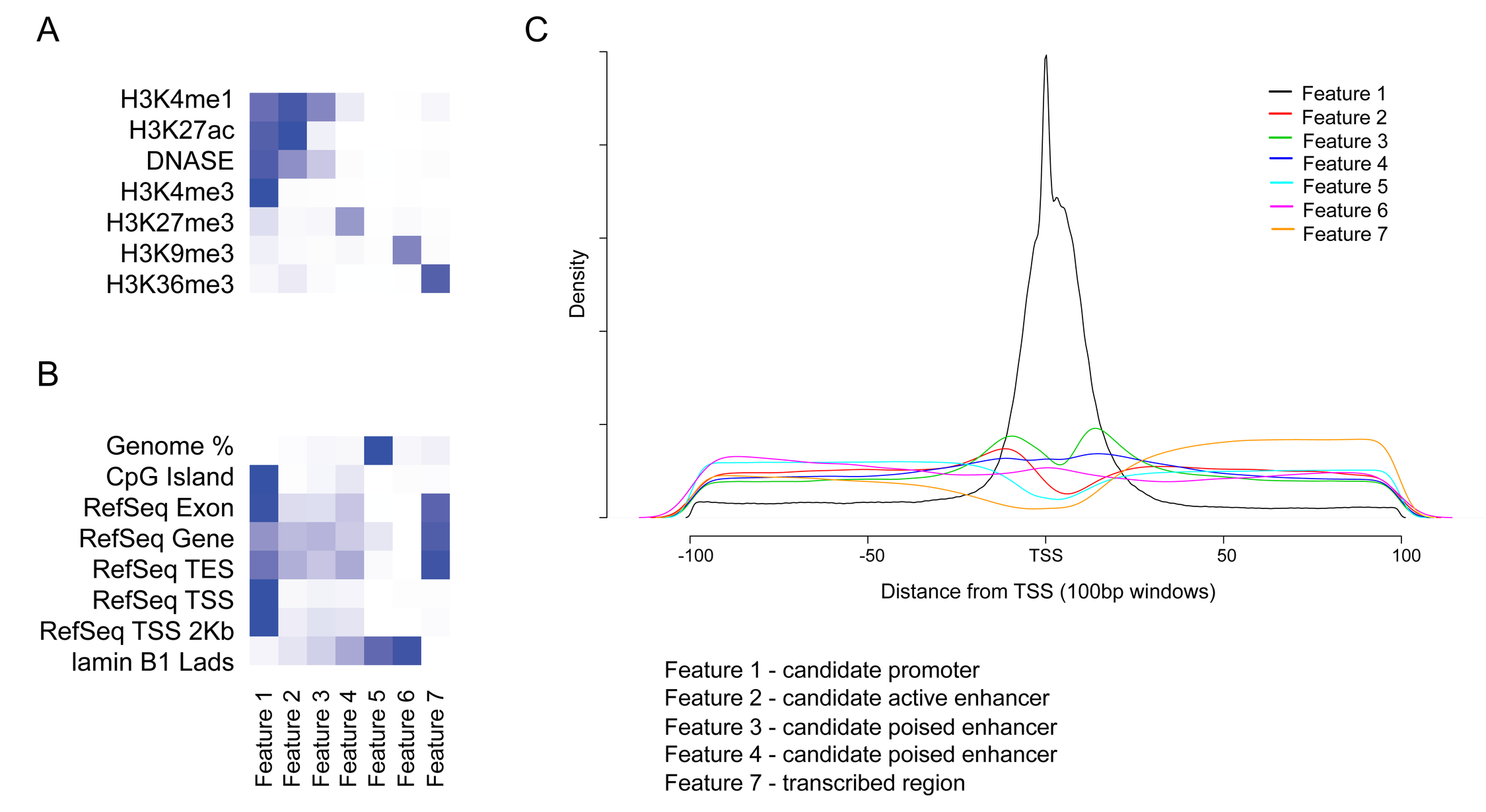

Supplement: S7 Fig — ChromHMM algorithm was used to define genomic annotations based on ChIP-seq tracks available for the placenta. Heatmap representing the enrichment for the different ChIP-seq mark in each feature (A). Heatmap representing the enrichment for previously defined genomic annotation in each feature (B). Density plot representing the enrichment for each feature in function of the distance from the transcription starting site (TSS) (C). (TIF) [file pgen.1007785.s007.tif]

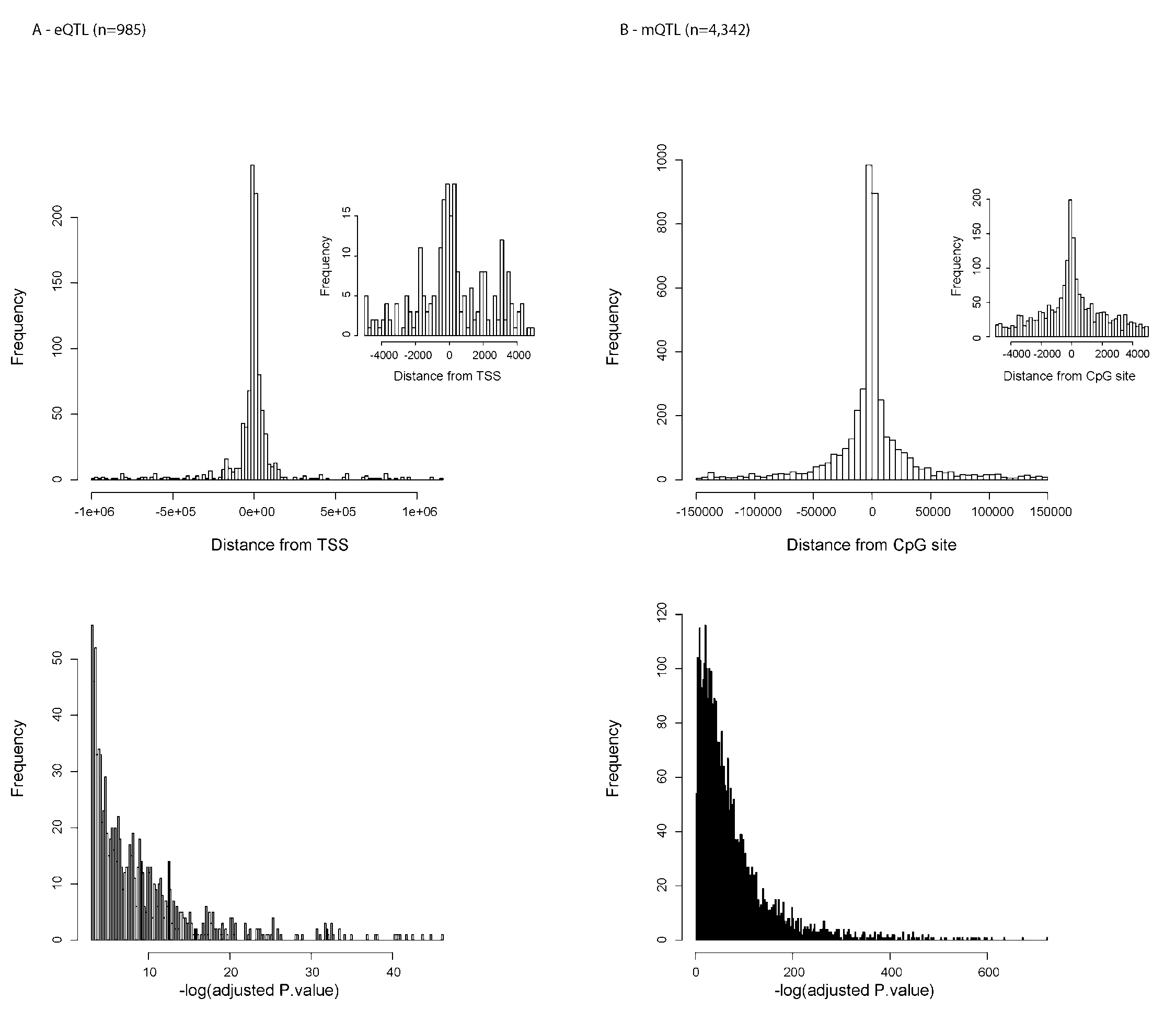

Supplement: S8 Fig — Histogram representing the distribution of associations from the transcription start site (TSS) for eQTL (A) and from the CpG site for mQTL (B). (TIF) [file pgen.1007785.s008.tif]

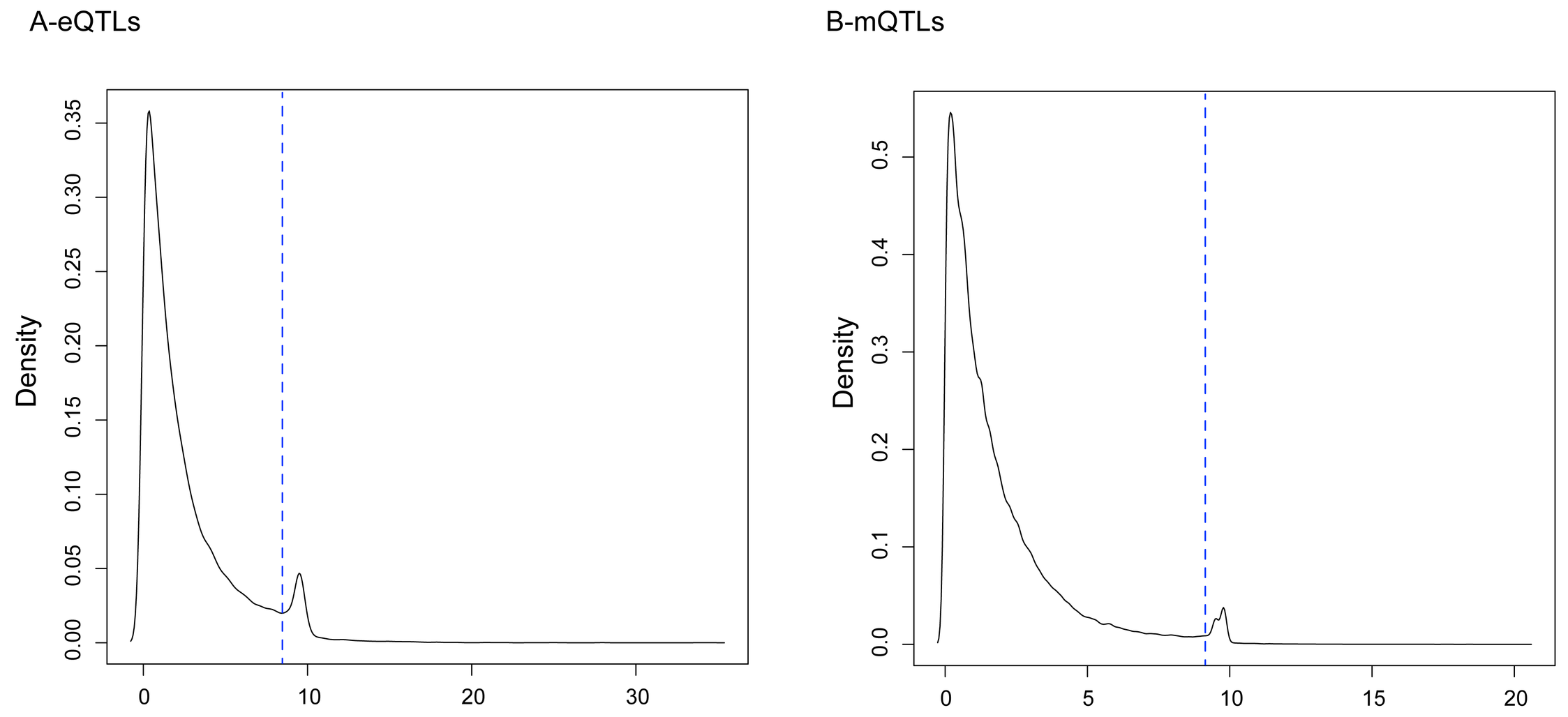

Supplement: S9 Fig — Representative density plot of the difference in binding affinity between the reference and alternative allele for eQTL (A) and mQTL (B) as defined by FIMO. Null differences and binding not reaching a p.value <0.0001 were excluded prior to analysis. (TIF) [file pgen.1007785.s009.tif]

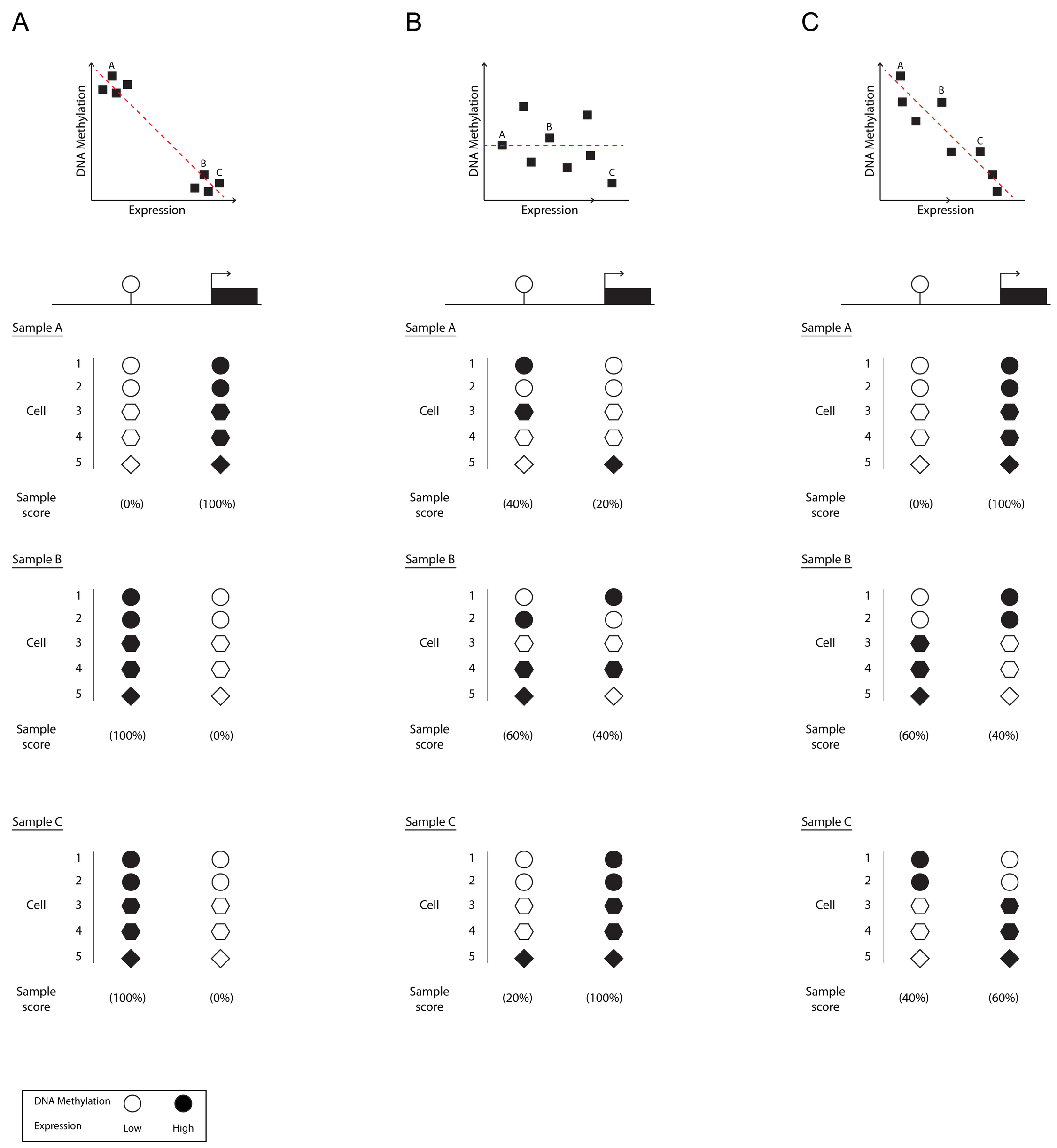

Supplement: S10 Fig — Scenario representing a correlation between CpG and gene which is not cell specific as DNA methylation and gene expression profiles are similar across the different cells of each sample. CpG is either fully or not methylated and expression is high or low. This scenario will be called as bimodal distribution (A). Scenario representing an absence of correlation between DNA methylation and gene expression (B). Scenario representing a correlation between CpG and gene which is cell specific as DNA methylation and gene expression profiles are variable across cell within each sample. This profile will be called a linear distribution (C). (TIF) [file pgen.1007785.s010.tif]

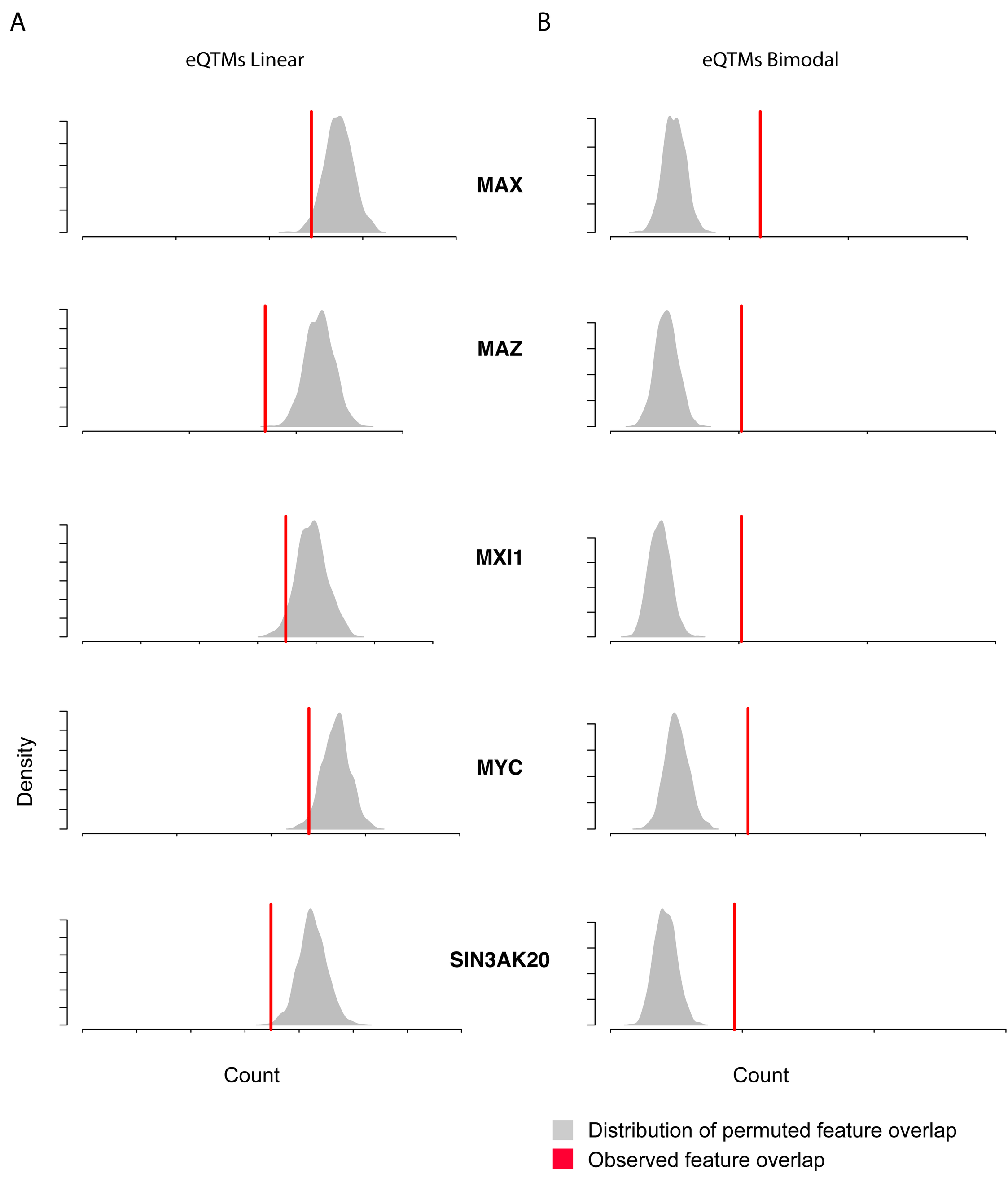

Supplement: S11 Fig — The density plots represent the distribution of overlaps between random sampling and the selected transcription factors binding site as defined by the ENCODE Factorbook repository when the red line illustrates the overlap value between candidate associations and the corresponding transcription factor binding site for linear (A) and bimodal (B) distributions. (TIF) [file pgen.1007785.s011.tif]

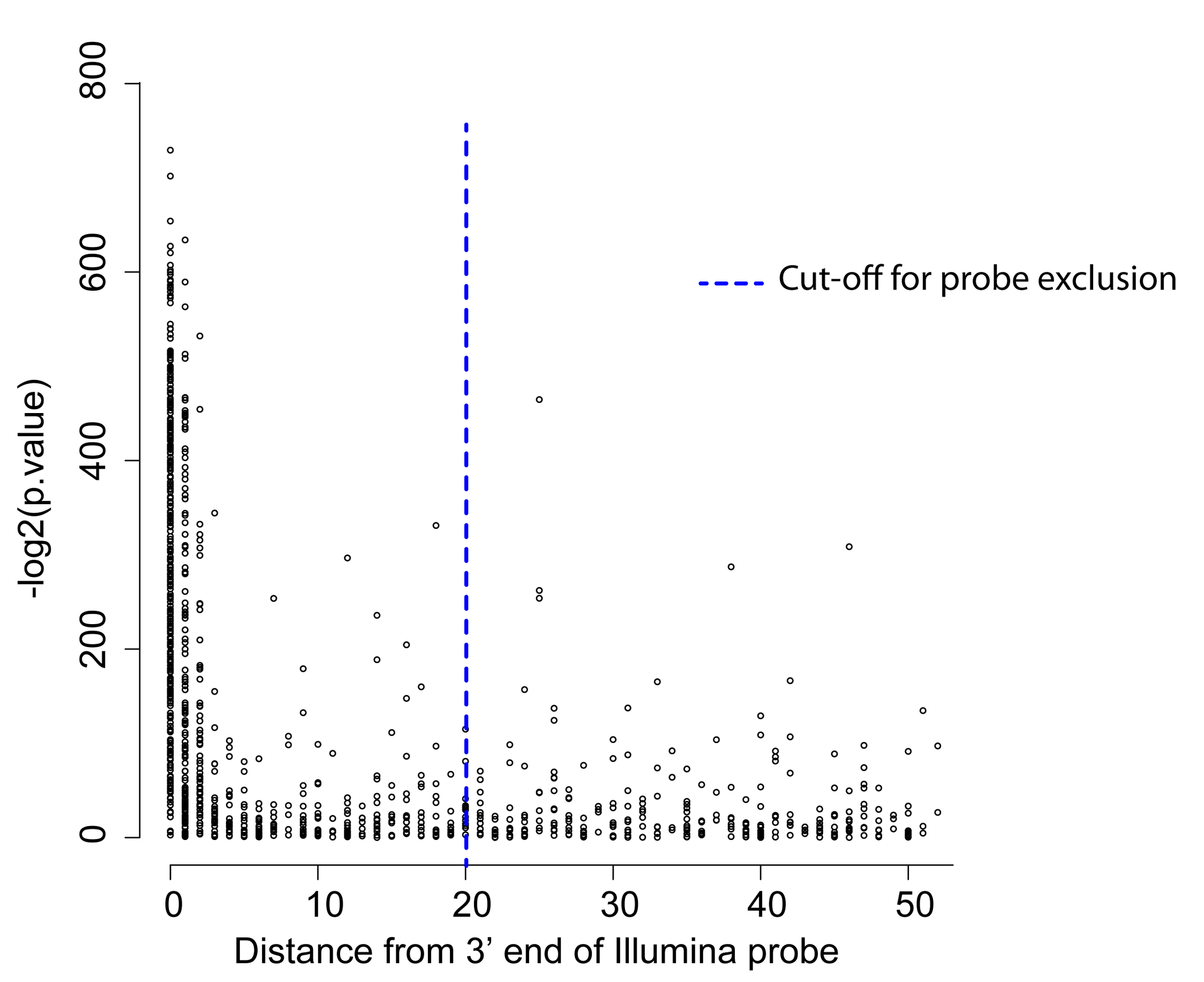

Supplement: S12 Fig — Scatter plot representing the enrichment for significant associations in function of distance from 3’end of Illumina probe. The blue dash line represents the cutoff use during our study. This cutoff was defined based on previous observations and on the decrease, enrichment observed in our analysis. (TIF) [file pgen.1007785.s012.tif]
